# Variation in costs to complete surgical training: a cross-sectional observation analysis

**DOI:** 10.1308/rcsann.2024.0088

**Published:** 2025-09-08

**Authors:** CL Eley, OP James, N Warren, C Carpenter, D Hanratty, RJ Egan, JD Barry, WG Lewis, on behalf of Wales’s Surgical Research Initiative Collaborative

**Affiliations:** ^1^ Aneurin Bevan University Health Board, UK; ^2^ Cardiff University, UK; ^3^ Cardiff and Vale University Health Board, UK; ^4^ Wales Institute for Minimal Access Therapy, UK; ^5^ Swansea Bay University Health Board, UK; ^6^ Swansea University, UK

**Keywords:** Surgical training, Cost, Education, Courses

## Abstract

**Introduction:**

Surgical training is expensive. The aim of this study was to quantify the costs surgical trainees are expected to pay related to the ten surgical specialties in a single Statutory Education Body (SEB).

**Methods:**

Intercollegiate Surgical Curriculum Programme (ISCP) and Joint Committee on Surgical Training (JCST) certification requirements including mandatory and highly recommended courses, related to specialty, along with professional registration and examination fees were estimated.

**Results:**

Assuming an uninterrupted eight-year training pathway from Core (CST) through Higher Specialty Training (HST) to Certification of Completion of Training, median cost of mandatory courses was £1,498 (interquartile range (IQR) £1,498–£1,998). Highly recommended courses increased median cost to £5,998 (£4,880–£6,840); £749.75 per year (py) (£610–£855). Annual individual study budget (£600) was exceeded in 30.7% of the total trainee cohort (*n*=309) by mandatory and recommended curriculum course cost (*n*=95). Examination fees and professional subscriptions further increased costs to a median £17,669.50 (£16,552–£18,512); £2,148.75 per person py (£2,069–£2,251.38). Cost varied related to specialty, with General Surgery associated with the most cost (>£21,000; £2,626 py) compared with Otolaryngology the least (£15,613; *p*<0.001).

**Conclusion:**

Surgical training expense varied by more than 33%. Mandatory and highly recommended courses exceeded SEB study budgets for almost one-third of trainees, with a theoretical fivefold study budget overspend. Trainees, trainers and schools of surgery alike should be aware of these costs when designing curricula and teaching programmes.

## Introduction

Learning is most effective with clear goals, evaluation and metric-defined credentials. High-quality simulated learning, long advocated by professions with high-risk job profiles in first-world 24-hour access economies, comes at high expense, both in terms of human effort and financial cost. Yet, education budgets are low, squaring to some two per cent of global healthcare spends.^1^

The price of attaining the mandatory credentials to obtain a Higher Surgical Training (HST) programme appointment in the UK, aligned to 2017 ISCP curriculum was high – estimated to be between £2,735 and £20,780 depending on surgical specialty (mean, £3,360) compared with medicine (£2,815) and anaesthesiology (£2,215).^2^ Moreover, after HST entry, costs to meet Completion of Certification of Training (CCT) credentials, set by the Joint Committee on Surgical Training (JCST) grow further and include UK General Medical Council registration, JCST registration fee, mandatory and highly recommended educational/simulation courses, professional examination fees, Royal College of Surgeons of England subscription fees, specialty society membership, learned society conference fees and medical indemnity insurance.

All the above is compounded by undergraduate-driven debt on qualification – estimated by the Association of Surgeons in Training (ASiT) to be more than £25,000, and rising, given the annual increased university tuition fees amassing to a minimum of £9,000 per annum (Wales), £9,250 (UK) and a reported average of £22,200 per year (py) for international students.^3–5^

The aims of this study were twofold: first, to quantify the costs surgical trainees are expected to pay related to 2021 Core Surgical Training (CST) and later HST curriculum, in the ten surgical specialties; second, to compare these professional expenses with the study leave budgets available to support trainees supplied from a Statutory Education Board (SEB) held with the local hospital trusts or health boards.

## Methods

Intercollegiate Surgical Curriculum Programme (ISCP) pan-specialty surgical curricula were reviewed with specific focus on the financial cost of training. This included mandatory and highly recommended courses related to specialty along with mandatory registration and examination fees from provider websites. Health Education Improvement Wales (HEIW) study budget was obtained (£600 per trainee py). Where clarification was needed, Training Programme Directors (TPD) were consulted to define highly recommended courses. Statistical analysis proper for nonparametric data (confirmed by Shapiro–Wilks test) was performed using SPSS Statistics version 27 (IBM Corp, Armonk, NY, USA).

## Results

Assuming an uninterrupted eight-year training pathway from Core Surgical Training (CST) / Specialty Training (ST) to Certification of Completion of Training (CCT), median cost for mandatory training courses was £1,498 (IQR £1,498–£1,998). Adding highly recommended courses, the median cost rises to £5,998 (£4,880–£6,840); £749.75 py (£610–£855) with 30.7% of trainees exceeding their study budget with mandatory and recommended courses (*n*=95 >£600 py). Added costs of further examinations and necessary professional subscriptions resulted in an estimated cost of £17,190 (£16,552–18,011), a median of £2,148.75 per person py (£2,069–2,251.38) (Table 1).

**Table 1:** Core surgical training costs, per trainee

	Cost per trainee (£)				
	Mandatory courses	Mandatory+recommended courses	Mandatory courses+additional training costs*	Mandatory, recommended courses+additional training costs*	Total per year*
Core Surgical Training	749	2,397	3,216	4,864	2,432.00

*Additional training costs: examination fee, professional registration. Further breakdown available in Appendix 1.

**Table 2:** Variation in surgical training costs, per trainee, CST – CCT (eight years)

	Median cost per trainee (£)				
	Mandatory courses	Mandatory+recommended courses	Mandatory courses+additional training costs	Mandatory, recommended courses+additional training costs*	Total per year*
General surgery	2,498	9,338	14,170	21,010	2,626.25
Paediatric surgery	2,844	7,113.80	14,516	18,805.80	2,350.73
Urology	749	6,840	12,421	18,512	2,314.00
Vascular	1,498	6,339	13,170	18,011	2,251.38
OMFS	1,498	6,395	13,170	18,011	2,251.38
Plastics	1,498	5,656	13,170	17,328	2,166.00
Trauma and orthopaedics	1,998	5,380	13,670	17,052	2,131.50
Neurosurgery	1,498	4,880	13,170	16,552	2,069.00
Cardiothoracic surgery	1,498	4,880	13,170	16,552	2,069.00
Otolaryngology	1,498	3,941	13,170	15,613	1,951.63

CCT = certification of completion of training; CST = core surgical training; OFMS = oral and maxillofacial surgery

*Additional training costs: examination fee, professional registration. Further breakdown available in Appendix 1.

**Figure 1:**
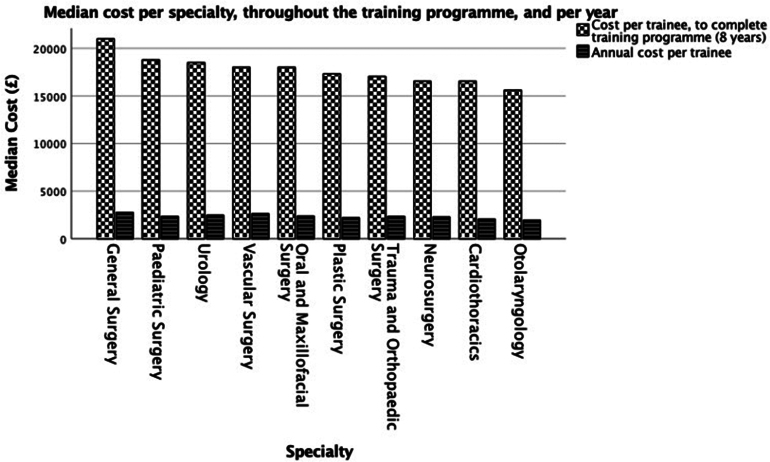
Median cost per specialty, throughout the training programme, and per year

General Surgical Trainees incur the highest cost py (£2,626) with a significant variation in estimated cost seen related to surgical specialty: General and Vascular Surgery proving the most expensive (>£21,000) compared with Otolaryngology the least expensive (£15,613; *p*<0.001) (Table 2, Figure 1). The total cost that a SEB would incur to cover mandatory courses for a cohort of 309 surgical trainees was £295,852, increasing to £1,127,174 including highly recommended courses (Table 3).

**Table 3:** Variation in surgical training costs, per specialty

	Cost per specialty (£)				
	Number of trainees	Mandatory courses	Mandatory+recommended courses	Mandatory courses+additional training costs*	Mandatory, recommended courses+additional training costs*
CST	105	78,645	251,685	337,680	510,720
Higher surgical training					
General surgery	66	99,434	442,106	706,964	1,049,636
Vascular	10	7,940	39,420	99,540	131,470
Urology	19	0	84,417	174,895	259,312
OMFS	12	8,988	47,976	119,448	158,436
Trauma and orthopaedics	51	63,699	152,133	624,393	712,827
Neurosurgery	6	4,494	14,898	59,724	70,128
Plastics	14	10,486	45,626	139,356	174,496
Paediatric surgery	2	4,190	5,283.60	22,600	27,883.60
Cardiothoracic surgery	7	5,243	17,381	69,678	81,816
Otolaryngology	17	12,733	26,248	169,218	182,733
Total		295,852	1,127,174	2,523,496	3,359,458

OFMS = oral and maxillofacial surgery

*Additional training costs: examination fee, professional registration. This no longer includes curriculum-specified conference attendance as this has been removed in the latest revision, although it still contributes to significant additional cost. Further breakdown available in Appendix 1.

## Discussion

‘And if you think education is expensive, wait until you see how much ignorance costs in the 21st century’.^3^

This is the first study to examine the costs associated with training since the start of the new JCST surgical curricula in 2021. The principal findings were that doctors aspiring to be surgeons spend several thousand pounds after graduation to obtain the mandatory credentials needed to complete training. Costs varied over 33%, ranging from £15,613 in Otolaryngology to north of £21,000 in General and Vascular Surgery (Table 2). General Surgical Training appeared most expensive, equating to £2,626 py – almost five-fold more than the assigned SEB training budget. Total costs included professional subscriptions, examination fees, and mandatory and highly recommended course charges.^4–14^ This amounted to a minimum of £7,562 in professional subscriptions (48.4%), £4,110 in examination fees (26.3%), and £1,498 in mandatory course charges, (9.6%) and £2,443 in highly recommended courses (15.6%), for Otolaryngology. Maximum total cost for General Surgery was split between £7,562 in professional subscriptions (34.2%), £4,110 in examination fees (18.6%), £2,498 in mandatory course charges (22.4%), with a further £6,840 in highly recommended course charges (19.9%).

Key factors that can influence the relative cost of medical training include, first, country and geographical location – different countries have different healthcare and education systems, which can affect tuition fees and living expenses; second, the type of Medical Programme – medical training can involve various levels of education, such as undergraduate medical school, postgraduate medical education (residency) and subspecialty fellowships. Each level may have different tuition fees and expense; third, public versus private institutions – public medical schools and training programmes may offer lower tuition fees for in-state or in-country residents compared with private institutions, but private schools may have higher tuition costs but may also offer more financial aid options; fourth, duration of training – length of medical training programmes varies; for example, residencies can last from three to seven years or more, depending on the specialty; fifth, supplies, equipment, and examination costs – some medical programmes include added costs for textbooks, laboratory supplies, medical equipment and licensing examinations; sixth, living expenses – the cost of living in the area where the medical training takes place can significantly affect overall expense, especially for longer training programmes; seventh, financial aid, scholarships, grants and other funding opportunities can alleviate the financial burden for some; eighth, subspecialty training – some medical specialties have higher associated costs because of the need for specialised resources.

Burnout among surgeons has become a well-recognised phenomenon. In 2020, Robinson *et al* reported that 59% of trainees showed prominent levels of burnout, highest at CST and attributed mostly to factors related to career development, including examinations and extensive curriculum demands.^15,16^ Arguably, the financial burden of these curriculum-associated demands – £4,864 in a two-year period – is likely to have contributed to career-related stressors (Table 1). Revision of the ISCP curriculum has seen a movement away from mandated RCS England courses, encouraging local delivery of courses relevant to specialty surgical development. The Newcastle Surgical Training Centre (NSTC), on the Freeman Hospital site, has been one of the leading UK training facilities, and has supplied advanced cadaveric simulated multiprofessional education for 15 years. Led by academic surgeons it also supplies research opportunities for the trial of new concepts and technologies in medicine in a safe environment. Collaboration between NHS, Academic partners (local universities and Royal Surgical Colleges), as well as industry alliances in the arenas of medicine, science and technology is important, and the emphasis on wet laboratories and fresh frozen cadavers is at the centre of their philosophy to supply a simulated environment as close to reality as practical.

The study has inherent limitations. The data pertain to only one SEB and may not apply to other SEBs with differing educational perspectives and financial resource. England and Scotland have reformed their procedures to offer more equitable access to funds. In 2018, Health Education England, now part of NHS England (NHSE), revised their study budget process by eliminating individually allocated capped values per trainee. Instead, any activity that supports curriculum outcomes, improves patient care or contributes to trainee development can be considered. Local educational providers manage a finite budget and must allocate funds carefully.^17^ This approach should address disparities in costs among more expensive specialties. However, with the removal of previously specified mandatory courses from the curricula, superficially suggesting a potential reduction in training-associated costs, the reality is more complex. Although no longer labelled as *mandatory*, they are still strongly recommended among the surgical community, meaning that, in practice, most trainees will still undertake them to remain competitive and properly prepared for day-one consultant-level clinical practice. As such, the overall financial burden on residents has not decreased. This reclassification has important implications for funding mechanisms where, with finite amounts of money, funding bodies prioritise courses explicitly required by the curriculum. This may paradoxically increase personal costs for trainees, now self-funding previously reimbursable training activities. NHS Scotland uses a Notional Annual Allocation based on the number of trainees in a programme, but this allocation serves as a reference rather than a monetary cap per trainee, with payments made upfront upon receipt of proof of purchase or payment.^18^ In Wales, study budgets are reimbursed retrospectively, meaning residents may be out of pocket for several months or longer before receiving their allocations. Moreover, this delay may be worsened by courses in high demand with long waiting lists. Like Wales, Northern Ireland provides annual study budgets per trainee to attend mandatory and nonmandatory courses, although this is more than twice the amount, valued at £1,250 per resident py.^19^ An ASiT national training survey in 2017 reported average costs of £20,000 to £26,000 per resident over the course of training, related to specialty. This included retrospectively collected individualised data relating to courses and conferences attended, as well as examinations and professional registration fees. The current study estimated costs since the revised ISCP curricula were introduced, which no longer defines the desired number of conferences attended. It remains clear that residents attend considerably more courses for their professional development than are mandated or highly recommended, at individual expense. Many residents supplement their learning with added subspecialty courses, and it must also be appreciated that the cost of surgical training begins well before CST to build a competitive application, showing a commitment to surgery as a specialty. Costs are further underestimated because of associated travel and accommodation expenses needed for attending courses outside of home geographical regions, especially challenging given the current cost-of-living crisis. Median number of MRCS (Membership Examination of the Surgical Royal Colleges of Great Britain and Ireland) attempts in a smaller subgroup was also examined to estimate the cost to the wider population. One-third of the current cohort needed three MRCS attempts or more. With the median pass rate for MRCS part A of 38.8%, repeated attempts are likely, resulting in further associated expense. Moreover, further added personal costs include undergraduate medical school tuition debt, travel, subsistence and additional professional examination fees.

Cost savings are plainly theoretically possible. The ISCP 2021 curricula tried to remove mandatory RCS England courses in support of locally, delivered and cheaper iterations. Generic curricula run by local SEBs may offer opportunities to address areas such as teaching, research and leadership, negating the need for formal cost-heavy courses run by outside national professional associations. For example, observation of Teaching (OoT) ISCP assessment can supply evidence through practical teaching and peer review, although curricula do not define the levels necessary to prove competence. Nevertheless, the highest value currency is perceived to be national specialty society or RCS England-certified courses supplying globally recognised credentials. In contrast, the study has strengths. It addresses the needs of a pan-specialty surgical group including core and higher surgical training, and engaged all key stakeholders, to inform the highly recommended postgraduate surgical courses. Moreover, it provides an up-to-date review of the minimum costs associated with the most recent iteration of ISCP-defined curricula and credentials believed mandatory for CCT.

## Conclusion

Because of the wide variety of medical training programmes and the differences between countries and institutions, it is difficult to supply an exact cost without specific detail. Aspiring medical professionals should research and compare the costs of various programmes they are interested in, consider their financial resource and explore available funding options to inform decisions about their medical education. Understanding the cost breakdown of training should help policymakers and healthcare institutions make informed decisions about resource allocations, curriculum development and training programme enhancements with targeted investments, long-term financial planning and sustainability measures at the forefront of modelling to ensure that surgical training programmes can meet the evolving needs of trainees and support a high future standard of surgical education.

## Competing interests

The authors declare no competing interests.

## Funding

The authors received no financial support for the research, authorship, and/or publication of this article.

## Supplementary Information

The online version contains supplementary material available at https://doi.org/10.1308/rcsann.2024.0088.
